# *Pseudomonas aeruginosa *inhibits *in-vitro Candida *biofilm development

**DOI:** 10.1186/1471-2180-10-125

**Published:** 2010-04-25

**Authors:** HMHN Bandara, JYY Yau, RM Watt, LJ Jin, LP Samaranayake

**Affiliations:** 1 Faculty of Dentistry, The University of Hong Kong, Oral Biosciences, 5/F, Prince Phillip Dental Hospital, 34, Hospital road, Sai Ying Pun, Hong Kong; 2 Faculty of Dentistry, The University of Hong Kong, The Department of Periodontology, 3/F, Prince Phillip Dental Hospital, 34, Hospital road, Sai Ying Pun, Hong Kong

## Abstract

**Background:**

Elucidation of the communal behavior of microbes in mixed species biofilms may have a major impact on understanding infectious diseases and for the therapeutics. Although, the structure and the properties of monospecies biofilms and their role in disease have been extensively studied during the last decade, the interactions within mixed biofilms consisting of bacteria and fungi such as *Candida spp*. have not been illustrated in depth. Hence, the aim of this study was to evaluate the interspecies interactions of *Pseudomonas aeruginosa *and six different species of *Candida *comprising *C. albicans*, *C. glabrata, C. krusei*, *C. tropicalis*, *C. parapsilosis*, and *C. dubliniensis *in dual species biofilm development.

**Results:**

A significant reduction in colony forming units (CFU) of *C. parapsilosis *(90 min), *C. albicans *and *C. tropicalis *(90 min, 24 h and 48 h), *C. dubliniensis *and *C. glabrata*, (24 h and 48 h) was noted when co-cultured with *P. aeruginosa *in comparison to their monospecies counterparts (P < 0.05). A simultaneous significant reduction in *P. aeruginosa *numbers grown with *C. albicans *(90 min and 48 h), *C. krusei *(90 min, 24 h and 48 h),*C. glabrata*, (24 h and 48 h), and an elevation of *P. aeruginosa *numbers co-cultured with *C. tropicalis *(48 h) was noted (P < 0.05). When data from all *Candida spp*. and *P. aeruginosa *were pooled, highly significant mutual inhibition of biofilm formation was noted (*Candida *P < 0.001, *P. aeruginosa *P < 0.01). Scanning Electron Microscopy (SEM) and Confocal Laser Scanning Microscopy (CLSM) analyses confirmed scanty architecture in dual species biofilm in spite of dense colonization in monospecies counterparts.

**Conclusions:**

*P. aeruginosa *and *Candida *in a dual species environment mutually suppress biofilm development, both quantitatively and qualitatively. These findings provide a foundation to clarify the molecular basis of bacterial-fungal interactions, and to understand the pathobiology of mixed bacterial-fungal infections.

## Background

In most natural environments, microorganisms exist predominantly as biofilms rather than as free floating planktonic cells [1]. A biofilm can be defined as a complex functional community of one or more species of microbes encased in extra cellular polymeric substances and, attached to one another or to a solid surface [2]. Biofilms can be composed of a single microbial species or more commonly, mixed species such as bacteria and fungi [3,4]. Perhaps the most studied example of the biofilm in humans is the dental plaque[5]. Microorganisms in the biofilm characteristically display a phenotype that is markedly different from that of their free floating counterparts [1]. For instance, they are resistant to antimicrobial agents in comparison to planktonic cells [6-8]. As more than 65% of biofilms with human microbial infections are caused by biofilms [5], there is an urgent need to understand biofilm behaviour.

The genus *Candida *comprises more than 150 pathogenic and nonpathogenic yeast species. Among these, *C. albicans, C. tropicalis, C. parapsilosis, C. krusei, C. kefyr, C. glabrata *and *C. guillermondii *are recognized as medically important pathogens [9]. *C. albicans *is the most prevalent yeast isolated from humans (47-75%) followed by *C. tropicalis *(7%), *C. glabrata *(7%), *C. krusei *(5%), *C. parapsilosis *(< 5%) and *C. guillermondii *(< 5%) [9]. Common Candidal habitats of humans include the gut, skin and mucosal surfaces, while one half of the human population carry *Candida *in their oral cavities[10].

*Pseudomonas aeruginosa *is an aerobic Gram-negative bacterium that causes community acquired infections, such as ulcerative keratitis, otitis externa, skin and soft tissue infections and, nosocomial infections including pneumonias, urinary tract infections, infections in surgical sites and burns [24,25]. Indeed, out of all nosocomial infections in different ethnic communities, 11-13.8% is found to be caused by *P. aeruginosa *[11-13]. United States Cystic Fibrosis Foundation Patients Registry (2004), has stated that 57.3% of all reported respiratory cultures contained *P. aeruginosa *indicating its important role in causing chronic and recurrent infections in cystic fibrotic patients [14]. Lee *et al *[15] have demonstrated that *P. aeruginosa *is the most commonly identified cause of septicemia in primary immunodeficiency and some 20% of bacteriaemia in acute leukemic patients [16,17]. Incidence of *P. aeruginosa *bacteriaemias in HIV affected patients is approximately 10 times higher than that of the normal population [18].

Pathogenic interactions between *C. albicans *and *P. aeruginosa *have recently been demonstrated by a number of groups [19,20]. The antifungal behaviour of *P. aeruginosa *against *Candida spp*. was first reported in early nineties by Kerr *et al *[20]. Subsequently others have shown that *P. aeruginosa *kills *C. albicans *by forming a dense film on fungal filaments, though, it neither binds nor kills the yeast-form of *C. albicans *[19]. Thein *et al *[21] have also reported that *P. aeruginosa *ATCC 27853 at a concentration gradient elicited a significant inhibition of *Candida albicans *biofilms.

Although, the structure and the properties of monospecies biofilms and their role in disease have been extensively studied during the last decade [22,23], the interactions within mixed biofilms consisting of bacteria and fungi including *Candida spp*. have not been studied in depth. Furthermore, the majority of the previous studies on interactions between *Candida *and bacteria in mixed biofilms have focused on *C. albicans *and there are only a few studies on non-albicans *Candida spp*. biofilms in a mixed species environment. Hence, the aims of this study were to evaluate the interactions of a reference laboratory strain of *P. aeruginosa *and six different *Candida *species, *C. albicans*, *C. glabrata*, *C. tropicalis*, *C. parapsilosis*, *C. dubliniensis*, and *C. krusei *in a dual species biofilms environment over a period of 2 days by both quantitative assays (Colony Forming Unit assay - CFU) and, qualitative evaluations using Scanning Electron Microscopy (SEM) and Confocal Laser Scanning microscopy (CLSM).

## Results

### Candida and *P. aeruginosa *dual species biofilm growth

After 90 min of biofilm development with *P. aeruginosa*, a significant, 57-88%, reduction in *Candida *counts was noted for *C. albicans *(57%, P = 0.005),*C. dubliniensis *(69%, P < 0.001),*C. tropicalis *(18%, P = 0.010) and *C. parapsilosis *(74%, P = 0.030) while *P. aeruginosa *did not impart such an effect on *C. glabrata *and *C. krusei *compared with the controls (Table 1). Conversely, after 90 min, a significant reduction in CFU of *P. aeruginosa *was observed in the presence of *C. albicans *(81%, P = 0.002) *C. krusei *(62%, P = 0.002) but not with the other four *Candida *species (Table 1).

**Table 1:** The mean CFU counts (± SD) of *Candida spp*. and *P. aeruginosa *from both monospecies and dual species biofilms at 90 min, 24 h and 48 h.

	Time interval	*Candida *CFU (10^6^) ± SD		P value	*P. aeruginosa *CFU (10^6^) ± SD		P value
					
		Control (MSB)	Test (DSB)		Control (MSB)	Test (DSB)	
***Candida*** ***albicans***	90 min	**12.60 ± 2.19**	**5.29 ± 1.52**	**0.005**	**3.44 ± 2.20**	**0.66 ± 0.69**	**0.002**
	24 h	**15.22 ± 3.31**	**5.00 ± 2.60**	**< 0.001**	876.89 ± 206.39	719.56 ± 266.53	0.200
	48 h	**31.89 ± 6.60**	**0.22 ± 0.44**	**< 0.001**	**1358.89 ± 323.59**	**922.22 ± 186.60**	**0.009**

***Candida krusei***	90 min	2.43 ± 1.46	2.71 ± 0.66	0.352	**7.32 ± 3.82**	**2.78 ± 1.29**	**0.003**
	24 h	3.39 ± 2.00	2.49 ± 0.73	0.301	**987.78 ± 341.79**	**583.33 ± 218.92**	**0.022**
	48 h	0.09 ± 0.14	0.22 ± 0.44	0.867	**140.00 ± 48.73**	**73.33 ± 35.71**	**0.010**

***Candida tropicalis***	90 min	**9.81 ± 3.05**	**3.87 ± 2.29**	**0.004**	1.42 ± 1.25	2.26 ± 0.71	0.070
	24 h	**27.67 ± 5.92**	**3.44 ± 1.59**	**< 0.001**	431.11 ± 66.23	471.11 ± 162.90	0.534
	48 h	**4.22 ± 2.05**	**0.00 ± 0.00**	**< 0.001**	**98.89 ± 75.74**	**351.11 ± 162.51**	**0.002**

***Candida parapsilosis***	90 min	**10.60 ± 6.71**	**1.26 ± 1.34**	**< 0.001**	4.87 ± 1.66	3.83 ± 2.31	0.228
	24 h	2.11 ± 2.32	0.78 ± 0.44	0.364	412.22 ± 208.55	277.78 ± 162.69	0.121
	48 h	0.89 ± 0.60	0.44 ± 0.73	0.120	183.33 ± 69.64	179.56 ± 50.02	0.859

***Candida glabrata***	90 min	10.81 ± 2.90	10.12 ± 3.97	0.659	9.91 ± 9.01	8.17 ± 5.03	0.691
	24 h	**35.78 ± 21.72**	**15.00 ± 21.08**	**0.024**	**328.89 ± 88.94**	**56.67 ± 15.81**	**< 0.001**
	48 h	**28.22 ± 17.14**	**0.11 ± 0.33**	**< 0.001**	**128.89 ± 69.54**	**28.89 ± 17.64**	**< 0.001**

***Candida dubliniensis***	90 min	**9.34 ± 3.21**	**2.94 ± 1.50**	**< 0.001**	9.83 ± 2.33	6.51 ± 4.35	0.070
	24 h	**5.81 ± 2.46**	**0.54 ± 0.88**	**< 0.001**	**878.89 ± 286.07**	**461.11 ± 142.78**	**0.003**
	48 h	0.00 ± 0.00	0.00 ± 0.00	1.000	97.78 ± 48.16	52.22 ± 50.94	0.056

P < 0.05 was considered statistically significant. Significant differences are shown in bold text.

However, after prolonged incubation for 24 hours, a significant, 58-91% reduction in the counts of *C. albicans *(67%, P < 0.001), *C. tropicalis *(88%, P < 0.001) *C. dubliniensis *(91%, P < 0.001) and *C. glabrata *(58%, P= 0.024) was noted in dual species biofilms with *P. aeruginosa *(Table 1) although *C. krusei *and *C. parapsilosis *counts were unaffected in comparison to the monospecies controls. On the other hand, mean CFU of *P. aeruginosa *decreased significantly in the presence of *C. krusei *(41%, P = 0.022), *C. dubliniensis *(48%, P = 0.003) and *C. glabrata *(83%, P < 0.001) after 24 h, while the other three *Candida *species had no significant effect on *P. aeruginosa *numbers at this time point (Table 1).

Most remarkable results were observed on further incubation for 48 hours, *C. albicans *(99%, P < 0.001), *C. tropicalis *(100%, P < 0.001) and *C. glabrata *(100%, P < 0.001) growth was almost totally suppressed in dual species biofilms with *P. aeruginosa *while the remaining *Candida *species were unaffected (Table 1). Simultaneously the mean CFU of *P. aeruginosa *decreased in co cultures of *C. albicans *(32%, P = 0.009) *C. krusei *(48%, P = 0.010), and *C. glabrata *(78%, P < 0.001). Conversely, *P. aeruginosa *counts significantly increased in the presence of *C. tropicalis *(72%, P = 0.002). Such an effect was not seen after 48 h with the two remaining *Candida *species,*C. dubliniensis *and *C. parapsilosis *(Table 1).

Despite these variable results, at different time intervals, when data from all *Candida spp*. were pooled and analyzed, a highly significant inhibition of *Candida *biofilm formation by *P. aeruginosa *(P < 0.001) and a simultaneous significant inhibition of *P. aeruginosa *biofilm development by *Candida *at all three time intervals (P < 0.01) was noted.

### Confocal laser scanning microscopy

**Figure 1:**
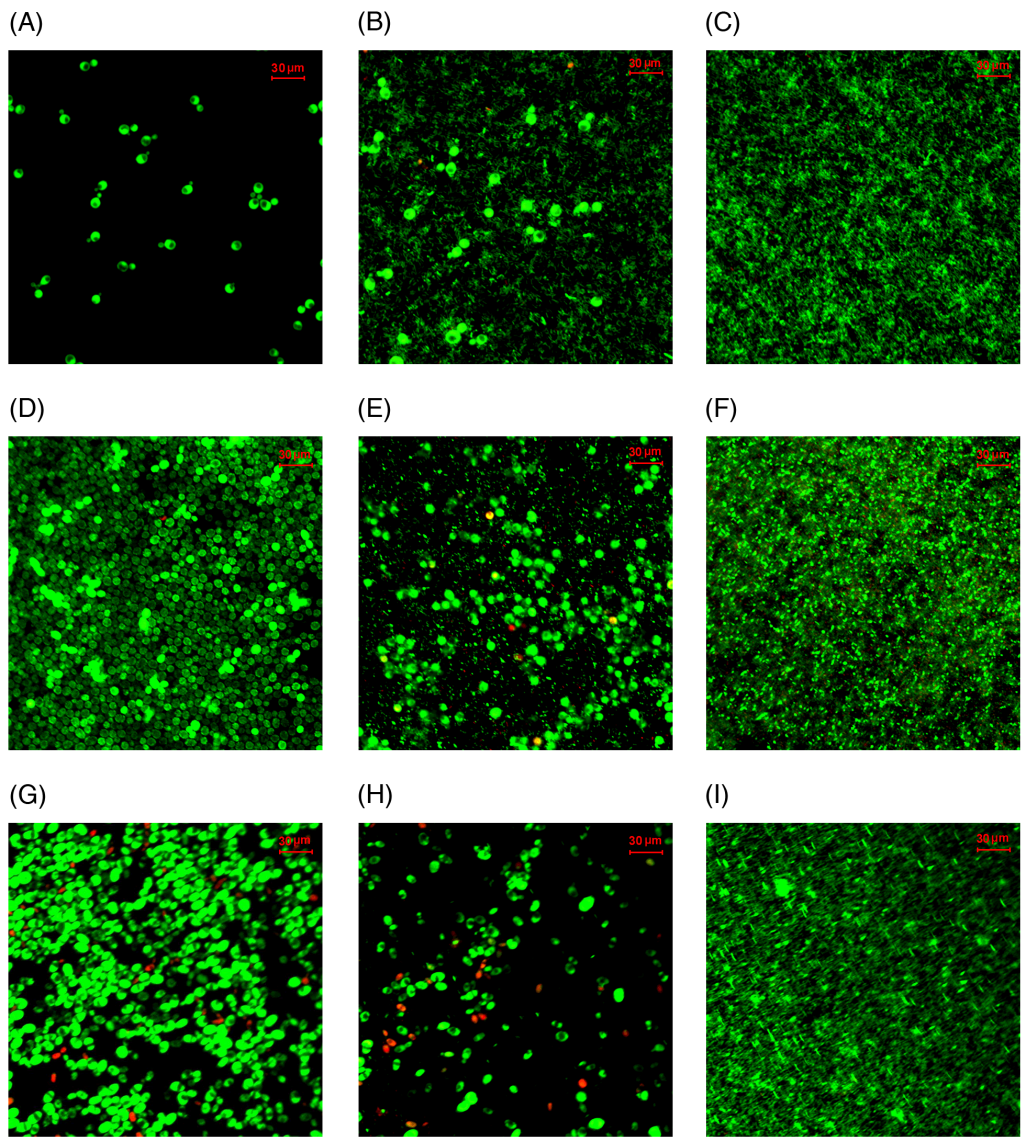
**CLSM images of monospecies (*Candida spp*. or *P. aeruginosa*) and dual species (*Candida spp*. and *P. aeruginosa*) biofilms**. (A). Adhesion of *C. albicans *for 90 min, (B). Adhesion of *C. albicans *and *P. aeruginosa *for 90 min, (C). Adhesion of *P. aeruginosa *for 90 min. Note the mutual inhibition of adhesion of both pathogens in dual species environment. (D) Initial colonization of *C. dubliniensis *for 24 h (E). Initial colonization of *C. dubliniensis *and *P. aeruginosa *for 24 h, (F). Initial colonization of *P. aeruginosa *for 24 h. Note the impaired biofilm formation after 24 h in the dual species biofilm due to mutual inhibition of these organisms. (G) Maturation of *C. tropicalis *for 48 h, (H). Maturation of *C. tropicalis *and *P. aeruginosa *for 48 h, (I). maturation of *P. aeruginosa *for 48 h. Note the altered and scant biofilm maturation in dual species biofilm as a result of mutual inhibition of *C. tropicalis *and *P. aeruginosa*.

CLSM with Live and Dead stain confirmed, in general, that *Candida spp*. and *P. aeruginosa *have mutually suppressive effects on each other at every stage of biofilm formation, in comparison to their monospecies counterparts. CLSM showed a reduction in both *Candida *and *P. aeruginosa *cells that were adherent after 90 min, confirming the data from CFU assay. Few dead *C. albicans *cells were also visible (Figure 1A, B and 1C).

In 24 h-dual species biofilms, mutual suppression of *C. dubliniensis *and *P. aeruginosa *was clearly seen, confirming CFU data. Thus, sparsely developed *C. dubliniensis *biofilm was seen with few dead cells in contrast to its dense monospecies biofilm, while *P. aeruginosa *numbers were greatly reduced compared to its monospecies counterpart (Figure 1D, E and 1F).

Similarly, after 48 h, sparsely distributed *C. tropicalis *blastospores were noted in dual species biofilms with few, scattered *P. aeruginosa *cells and a scant biofilm once again confirming the aforementioned quantitative CFU findings. Some dead cells and cellular debris were also observed compared to dense monospecies biofilm growth of *C. tropicalis *control (figure 1G, H and 1I).

### Scanning Electron Microscopy

**Figure 2:**
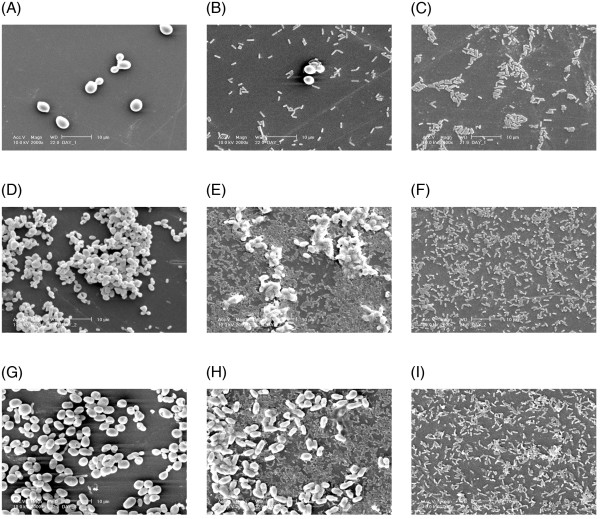
**SEM images of monospecies (*Candida spp*. or *P. aeruginosa*) and dual species (*Candida spp*. and *P. aeruginosa*) biofilms**. (A). Adhesion of *C. albicans *for 90 min, (B). Adhesion of *C. albicans *and *P. aeruginosa *for 90 min, (C). Adhesion of *P. aeruginosa *for 90 min. Note that there are few *C. albicans *blastospores with some degrading cells and few cells of *P. aeruginosa *in dual species biofilm in compared to monospecies counterparts. (D) Initial colonization of *C. glabrata *for 24 h (E). Initial colonization of *C. glabrata *and *P. aeruginosa *for 24 h, (F). Initial colonization of *P. aeruginosa *for 24 h. Note that *C. glabrata *is less in number with altered morphology while thin and scant biofilm was formed in the presence of *P. aeruginosa*. (G) Maturation of *C. tropicalis *for 48 h, (H). Maturation of *C. tropicalis *and *P. aeruginosa *for 48 h, (I). Maturation of *P. aeruginosa *for 48 h. Note the reduction in number and altered morphology of *C. tropicalis *in dual species biofilm.

Although species specific growth variations could be noted, in general, single species biofilms of all *Candida *species demonstrated profuse growth and dense colonization of the substrate on SEM observation (Figure 2). After 90 min, i.e. adhesion phase, the control monospecies *Candida *and *P. aeruginosa *cells were seen well-adherent and uniformly distributed on the polystyrene surface. Yeast blastospores were seen aggregated either in pairs or clumps with some budding yeasts. During 24 h of initial colonization phase, monospecies biofilms of both *Candida *and *P. aeruginosa *showed increased numbers of cellular layers with recognizable extracellular matrix. After 48 h, the single species biofilms of both pathogens were relatively thick and multilayered, although the extracellular matrix was scarcely visible.

However, on visual examination by SEM, dual species biofilms demonstrated reduction of yeast blastospores at each stage of biofilm formation compared to their monospecies counterparts. Specially in the maturation stage at 48 h, this reduction was marked and recognizable. The former biofilms were also less dense than the monospecies controls, and demonstrated few layers of cells, profuse cellular debris, together with degrading and morphologically altered yeast cells. Interestingly, most of the bacteria were seen attached to the blastospores (figure 2E and 2H). Bacterial density varied in the presence of different *Candida *species at different time intervals. In general, *P. aeruginosa *distribution was scanty and nondescript in the dual species environment (Figure 2B, E and 2H).

Quantitatively, smaller numbers of clumped *C. albicans*, together with some degrading blastospores, were observed with *P. aeruginosa *at the end of the adhesion phase, and the latter was also lesser in number compared to the monospecies variant (Figure 2A, B and 2C). A thin, scant biofilm, formed by a lesser numbers of morphologically altered *C. glabrata *was noted after initial colonization (Figure 2C, D and 2E). Furthermore, a few, morphologically altered blastospores of *C. tropicalis *were visible in mature dual species biofilm with *P. aeruginosa *at 48 h. In contrast, *P. aeruginosa *demonstrated thicker biofilms in the presence of *C. tropicalis*, compared to its mature monospecies variant (Figure 2G, H and 2I).

## Discussion

*Candida *and *P. aeruginosa *are major pathogens of device-associated nosocomial infections for virtually all types of indwelling devices [24]. It has also been stated that, the coexistence of *Pseudomonas *spp. and *C. albicans *in elderly is a potential indicator of high risk for pneumonia [25]. Recent experimental studies have identified similarities in environmental factors such as its physical and chemical nature where *P. aeruginosa *and *C. albicans *coexist [26]. As a result, these two microorganisms have become obvious candidates and models for the study of biofilm infections in order to develop potential methods for the control of device-associated nosocomial infections[24].

The principle aim of this study was to evaluate the qualitative and quantitative effects of *P. aeruginosa *on various stages of *in-vitro *biofilm formation of six different *Candida *species. Our results indicate that both *Candida *and *P. aeruginosa *mutually inhibit biofilm development to varying degrees at different stages of biofilm formation. However, the most important conclusion of our study is the ability of *P. aeruginosa *to almost totally inhibit *C. albicans, C. glabrata *and *C. tropicalis *in 48 h biofilms.

Using a CFU assay, we report here for the first time, the quantitative effect of *P. aeruginosa *on biofilm formation of six different *Candida *species in a time dependant manner. Our results indicate that *P. aeruginosa *had significant inhibitory effects on several *Candida spp*. such as, *C. albicans, C. dubliniensis, C. tropicalis*, and *C. parapsilosis*. In contrast, El-Azizi [27] found that *Pseudomonas *had no significant effect on *C. albicans *adhesion and biofilm growth, regardless of adding preformed *Pseudomonas *biofilms to *C. albicans *or vice versa. As there appeared to be differences in the mode of attachment of *P. aeruginosa *to yeast form of *C. albicans *or its filamentous form [28], mixed biofilm development between these two organisms could be a function of these characteristics.

Thein *et al *[21] from our group reported that, on prolong incubation for 2 days, *P. aeruginosa *ATCC 27853 at a concentration gradient, elicited a significant inhibition of *C. albicans *biofilm with a mean reduction in the number of viable Candidal cells ranging from 38% to 81%. Our results extend their work further and indicate that *P. aeruginosa *suppresses several other *Candida *species on incubation for upto two days, for instance, *C. dubliniensis *at 24 h and,*C. albicans, C. glabrata *and *C. tropicalis *both at 24 h and 48 h. In this context, Kaleli *et al *[29] investigated the anticandidial activity of 44 strains of *P. aeruginosa*, isolated from a number of specimens of intensive care patients, against four *Candida *species (*C. albicans, C. tropicalis, C. parapsilosis *and *C. krusei*) by a cross streak assay and subcutaneous injections of both bacterial and fungal suspensions into mice. They found that all *Pseudomonas *strains tested inhibited all four *Candida *species to varying degrees. *C. albicans *and *C. krusei *were the most inhibited while *C. tropicalis *were the least [29]. In contrast, our data show that the most significant inhibition elicited by *P. aeruginosa *was *C. albicans *and *C. tropicalis *while, the least was *C. krusei*. Grillot *et al *[30] observed complete or partial inhibition of *C. albicans, C. tropicalis, C. parapsilosis *and *C. glabrata *by *P. aeruginosa *in pure and mixed blood cultures using *in-vitro *yeast inhibition assays and suggested that preclusion of yeast recovery from blood cultures in mixed infections, such as polymicrobial septicemia, may be due to suppression of yeast by *P. aeruginosa*. In another study Kerr [20] demonstrated that nine *Candida *species, out of eleven tested, including *C. krusei, C. kefyr, C. guillermondii, C. tropicalis, C. lusitaniae, C. parapsilosis, C. pseudotropicalis, C. albicans *and *Torulopsis glabrata *were suppressed by *P. aeruginosa*. This *in-vitro *susceptibility test was performed with ten different strains of *P. aeruginosa *obtained from the sputum of three patients. Moreover, *C. albicans *was the most susceptible to growth inhibition followed by *C. guillermondii *and *T. glabrata*. Hockey *et al *[31], using an *in-vitro *model, studied the interactions of six different bacteria including *P. aeruginosa *and three pathogenic *Candida *species (*C. albicans, C. tropicalis*, and *T. glabrata*). The results of this study indicated that all three *Candida *species were suppressed by *P. aeruginosa *and *Klebsiella pneumoniae *in culture media. They further explained that this inhibition could be due to nutritional depletion and secretion of bacterial toxins. Interestingly, our results in general, concur with the foregoing findings as we too noted a significant inhibitory effect of *P. aeruginosa *on *C. albicans*, *C. tropicalis *and *C. parapsilosis *at different stages of their biofilm development. However, it should be emphasized that all of the foregoing studies were done in mixed culture media and our results are derived from a biofilm model.

In addition, as our study was bidirectional, we noted that some of the *Candida *species also suppressed *P. aeruginosa *during adhesion, initial colonization and maturation in dual species environment. Particularly, *C. albicans *at 90 min, *C. dubliniensis *at 24 h,*C. albicans, C*. *krusei*, and *C. glabrata *at both 24 and 48 h and *C. tropicalis *at 48 h.

Therefore, our results further authenticate the mutual inhibition and aggregation of certain *Candida spp*. and *P. aeruginosa*. Further works with multiple strains of *Candida *from different species are requested to confirm the species specificity of these findings.

Ultrastructural views of both monospecies and dual species biofilms confirmed the results obtained from quantitative assays. Basically, all monospecies biofilms of both *Candida *and *P. aeruginosa *demonstrated a well organized biofilm structure where yeasts were uniformly distributed with minimal amounts of extracellular substance, dead cells and cellular debris. The mature monospecies biofilms showed a characteristically thick layered structure. In contrast, dual species biofilms consisted of less dense *Candida *and *P. aeruginosa *growth, larger numbers of clumped cells, dead cells and cellular debris demonstrating the mutual inhibitory effect of these two pathogens in a dual species environment.

## Conclusions

In conclusion, this study, principally focused on the interactions of *Candida spp*. and *P. aeruginosa *during different stages of biofilm development, indicates the latter pathogens have significant mutual growth inhibitory effect at various stages of biofilm development in a dual species environment. It is also evident that there are species specific variations of this modulatory effect. Further work is necessary to clarify the molecular basis of these bacterial-fungal interactions, and to understand the pathobiology of mixed bacterial-fungal infections.

## Methods

### Experimental design

The study comprised a series of experiments to evaluate the combined effect of each of the aforementioned six *Candida spp*. and *P. aeruginosa *on their biofilm formation, quantitatively with CFU assay and qualitatively with CLSM and SEM, at three different time intervals, 90 min, 24 h and 48 h.

### Microorganisms

The following Reference laboratory strains of both *Candida *and *P. aeruginosa *were used, *Candida albicans *ATCC 90028, *Candida glabrata *ATCC 90030, *Candida tropicalis *ATCC 13803, *Candida parapsilosis *ATCC 22019, *Candida krusei *ATCC 6258, *Candida dubliniensis *MYA 646 and *Pseudomonas aeruginosa *ATCC 27853. The identity of each organism was confirmed with the commercially available API 32 C (for *Candida *strains) and API 20 E (for *P. aeruginosa*) identification systems (Biomérieux, Mercy I'Etoile, France). All isolates were stored in multiple aliquots at -20°C, after confirming their purity.

### Growth media

Sabouraud Dextrose Agar (SDA), Yeast Nitrogen Base (YNB) solution supplemented with 100 mM glucose were used for culturing *Candida *species while, Blood agar, MacConkey agar and Tryptic Soy Broth (TSB) were utilized for *P. aeruginosa *culture.

### Microbial inocula

Prior to each experiment, *Candida spp*. and *P. aeruginosa *were subcultured on SDA and blood agar, respectively for 18 h at 37°C. A loopful of the overnight *Candida *growth was inoculated into YNB medium, *P. aeruginosa *into TSB medium and, incubated for 18 h in an orbital shaker (75 rpm) at 37°C. The resulting cells were harvested, washed twice in Phosphate Buffered Saline (PBS, pH 7.2) and resuspended. Concentrations of *Candida spp*. and *P. aeruginosa *were adjusted 1×10^7 ^cells/mL by spectrophotometry and confirmed by hemocytometric counting.

### Biofilm Formation

*Candida *biofilms were developed as described by Jin *et al *[32] with some modifications. Commercially available pre-sterilized, polystyrene, flat bottom 96-well microtiter plates (IWAKI, Tokyo, Japan) were used. At first, 100 μL of standard cell suspensions of *Candida spp*. and *P. aeruginosa *(10^7^organisms/mL, 1:1 ratio) were prepared and transferred into selected wells of a microtiter plate, and incubated for 90 min at 37°C in an orbital shaker at 75 rpm to promote microbial adherence to the surface of the wells. Hundred microliters of monospecies controls of both *Candida spp*. and *P. aeruginosa *were inoculated in an identical fashion. After the adhesion phase, the cell suspensions were aspirated and each well was washed twice with PBS to remove loosely adherent cells. A total of 200 μL of TSB was transferred to each well and the plate reincubated for 24 h and for 48 h, and wells washed twice and thrice at respective time intervals with PBS to eliminate traces of TSB. The bacterial/fungal interactions were studied at 90 min, 24 h, and 48 h time intervals as follows.

### Quantitative analyses

#### Spiral plating and colony forming units assay (CFU)

At the end of the adhesion (90 min), colonization (24 h) and maturation (48 h) phases, 100 μL of PBS was transferred into each well and the biofilm mass was meticulously scraped off the well-wall using a sterile scalpel [32]. The resulting suspension containing the detached biofilm cells was gently vortexed for 1 min to disrupt the aggregates, serially diluted, and inoculated by a spiral plater on SDA for *Candida spp*. and, on MacConkey agar for *P. aeruginosa*. The resulting CFU of yeasts and bacteria were quantified after 48 h incubation at 37°C. Each assay was carried out in triplicate at three different points in time.

#### Qualitative analyses

Confocal Laser Scanning Microscopy (CLSM) [33] and Scanning Electron microscopy (SEM) were used to observe the ultrastructure of *Candida *and *P. aeruginosa *biofilms.

#### Confocal Laser Scanning Microscopy

Commercially available presteriled flat bottom six well plates (Iwaki, Japan) and presteriled plastic coupons (Thermanox plastic cover slips, Nulge Nunc International, Rochester, NY, USA) [34] were used to prepare biofilms as described above. Presteriled coupons were placed in wells of a 6-well plate, suspensions of monospecies or dual species added and the plate incubated for 90 min (the adhesion phase) in an orbital shaker (75 rpm) at 37°C. Thereafter, the supernatant was removed, washed twice with PBS, fresh TSB added and incubated for 24 hours (initial colonization) or 48 hours (maturation) under same environmental conditions. At the end of each time interval, the prewashed coupons were stained with Live and Dead stain (Live/Dead BacLight Bacterial Viability kit, Invitrogen, Eugene, USA). The biofilm architecture was then analyzed by fluorescent microscopy (using Confocal Laser Scanning Microscope).

#### Scanning Electron Microscopy

For SEM, we developed single species biofilms (*Candida *alone and *P. aeruginosa *alone) as well as *Candida *and *P. aeruginosa *mixed biofilms on custom made, tissue culture treated, polystyrene coupons as described above. At 90 min, 24 h, 48 h, selected coupons were removed from the wells, washed twice with PBS and placed in 1% osmium tetroxide for 1 h. Samples were subsequently washed in distilled water, dehydrated in increasing concentrations of ethanol (70% for 10 min, 95% for 10 min, and 100% for 20 min), and air dried in a desiccator prior to sputter coating with gold. Then the specimens were mounted on aluminium stubs, with copper tape, coated with gold under low-pressure with an ion sputter coater (JEOL JFC1 100: JEOL, Tokyo, Japan). The surface topographies of the biofilm were visualized with a scanning electron microscope (Philip XL30CP) in high-vacuum mode at 10 kV, and the images processed.

### Statistical analysis

Statistical analysis was performed using SPSS software (version 16.0). Mann--Whitney U test was performed to compare the significant differences between control and each test sample of the bacterial/Candidal biofilm. Data from all *Candida spp*. and *P. aeruginosa *analyses at different time points were pooled, and evaluated using Wilcoxon matched-pairs test. A P-value of < 0.05 was considered statistically significant.

## Abbreviations

CFU: Colony Forming Unit; SEM: Scanning Electron Microscopy; CLSM: Confocal Laser Scanning Microscopy; SDA: Sabouraud Dextrose Agar; YNB: Yeast Nitrogen Base; TSB: Tryptic Soy Broth; PBS: Phosphate Buffered Saline.

## Authors' contributions

LPS, LJJ, RMW and HMHNB conceived this research. HMHNB and JYYY designed and performed the experiments. HMHNB, LPS, LJJ contributed in data analysis and interpretation. HMHNB drafted the manuscript and it was reviewed by LPS, LJJ, RMW and JYYY. All authors read and approved the final manuscript.
